# Inhibition of CCAR1, a Coactivator of β-Catenin, Suppresses the Proliferation and Migration of Gastric Cancer Cells

**DOI:** 10.3390/ijms18020460

**Published:** 2017-02-21

**Authors:** Te-Sheng Chang, Kuo-Liang Wei, Chung-Kuang Lu, Yi-Hsing Chen, Ying-Tung Cheng, Shui-Yi Tung, Cheng-Shyong Wu, Ming-Ko Chiang

**Affiliations:** 1 Department of Gastroenterology and Hepatology, Chang Gung Memorial Hospital, Chiayi 61303, Taiwan; 2 College of Medicine, Chang Gung University, Taoyuan 33302, Taiwan; 3 Department of Life Science, National Chung Cheng University, Chiayi 62102, Taiwan

**Keywords:** gastric cancer, CCAR1, Wnt signaling, β-catenin, metastasis

## Abstract

The aberrant activation of Wnt signaling has been implicated in a variety of human cancers, including gastric cancer. Given the current hypothesis that cancer arises from cancer stem cells (CSCs), targeting the critical signaling pathways that support CSC self-renewal appears to be a useful approach for cancer therapy. Cell cycle and apoptosis regulator 1 (CCAR1) is a transcriptional coactivator which has been shown to be a component of Wnt/β-catenin signaling, and which plays an important role in transcriptional regulation by β-catenin. However, the function and clinical significance of CCAR1 in gastric cancer have not been elucidated. Here, we show that elevated CCAR1 nuclear expression correlates with the occurrence of gastric cancer. In addition, RNAi-mediated CCAR1 reduction not only suppressed the cell growth and increased apoptosis in AGS and MKN28 cells, but also reduced the migration and invasion ability of these cells. Furthermore, an in vivo xenograft assay revealed that the expression level of CCAR1 was critical for tumorigenesis. Our data demonstrates that CCAR1 contributes to carcinogenesis in gastric cancer and is required for the survival of gastric cancer cells. Moreover, CCAR1 may serve as a diagnostic marker and a potential therapeutic target.

## 1. Introduction

Gastric cancer is the fourth most common cancer and the second most common cause of cancer death after lung cancer, because of the poor prognosis of patients [1,2]. In Taiwan, gastric cancer is the sixth most common cause of cancer death [3]. Generally, gastric carcinogenesis can be viewed as a multi-step process involving *Helicobacter pylori* (*H. pylori*) infection, genetic alterations, and other risk factors that cause cells to progressively transform into cancer [4]. Currently, the regulatory mechanisms of progression in gastric cancer are still poorly understood.

Many reports support the concept that tumors contain a small subset of cells, the “cancer stem cells (CSCs)”, which exhibit a self-renewal capacity and which are responsible for tumor maintenance and metastasis. In the intestine, mutations in long-lived stem cells (*Lgr5^+^*), expressing leucine-rich repeat-containing G-protein coupled receptor 5), located at the crypt bottoms, are believed to result in intestinal cancer [5]. Similarly, *Lgr5* is also expressed in the bottom of gastric glands, and lineage tracing experiments show that the entire gastric gland is derived from *Lgr5*^+^ cells [6]. It is very likely that, due to the accumulation of mutations, *H. pylori* infection, epigenetic changes, and genetic alteration, dysregulation of the signaling pathways that control these *Lgr5*^+^ stem cells will give rise to gastric cancer.

Since CSCs are expected to be similar to the original tissue stem cells, the signaling that regulates the survival and growth of tissue stem cells is consequently becoming a new drug target for the treatment of CSCs. Previous studies have shown that Wnt signaling plays an important role in the self-renewal and maintenance of stem cells [7]. The Wnt signaling transduction cascades consist of a series of molecules that are regulated by different effector molecules. In particular, the canonical Wnt signaling is regulated by controlling the stability of its core component, β-catenin. β-catenin normally forms a stable complex with the cell adhesion molecules of the cadherin family, involved in the formation of adherens junctions. In the absence of Wnt signals, free cytosolic β-catenin is captured by a destruction complex containing Gsk3β, Axin, and Apc, where it is first phosphorylated, then ubiquitinated, on its N-terminal region, and subsequently targeted to proteosomes for its destruction. On the other hand, when the Wnt ligands are present, the destruction complex is disassembled through phosphorylation of Lrp5/6 and the binding of Axin to Lrp, which prevents β-catenin degradation [8,9]. Free β-catenin can then enter the nucleus, where it binds to the Tcf/Lef family and activates transcription of Wnt downstream target genes, such as *cyclin D1*, *c-myc*, *MMP-7*, *Lgr5*, and *CD44* [10,11]. Indeed, the accumulation of β-catenin in the nucleus, a sign of activated Wnt signaling, has been detected by immunohistochemical staining in a number of tumors, including colorectal, lung, breast, cervical, skin, and liver [12]. In addition, mutations affecting the components of the Wnt signaling pathway are frequently detected in various types of cancer [13,14]. In particular, mutations in the *Apc* gene were found in approximately 85% of colorectal cancer cases [15], and activating β-catenin mutations that affect its phosphorylation by Gsk3β, have been identified in 50% of colon cancers that have wild-type *Apc*. Considering that the stomach and intestine share the same origin during development, and their adult stem cells express the same specific marker (*Lgr5*), it is likely that the Wnt signaling pathway has similar impacts on the development of gastric cancer and the development of colorectal cancer.

Cell cycle and apoptosis regulator 1 (CCAR1/CARP-1) was initially identified as a protein that was required for apoptosis signaling by a retinoid (CD437), as well as the chemotherapeutics Adriamycin (ADR) and Etoposide in the human breast cancer cells [16]. Moreover, CCAR1 has been shown to be involved in cell-growth inhibitory and apoptosis-promoting effects, caused by the inhibition of the epidermal growth factor receptor [17]. However, CCAR1 has also been shown to be essential for estrogen-induced gene expression and the estrogen-dependent growth of human breast cancer cells [18]. Recently, a study revealed that CCAR1 associates with β-catenin and enhances the transcriptional activation of β-catenin target genes in colon cancer cells [19]. Considering that Wnt signaling supports the maintenance of stomach stem cells, we speculate that CCAR1 may play a major role in the tumorigenesis and metastasis of gastric cancer. Here, we show that CCAR1 is required for the survival of gastric cancer cells, and that the suppression of CCAR1 results in a decreased invasive character of gastric cancer cells. The results of this study may provide us with a potentially valuable therapeutic target for treating gastric cancer patients.

## 2. Results

### 2.1. Suppression of CCAR1 Reduces the Expression of Wnt Signaling Target Genes and Inhibits Gastric Cancer Cell Growth

**Figure 1:**
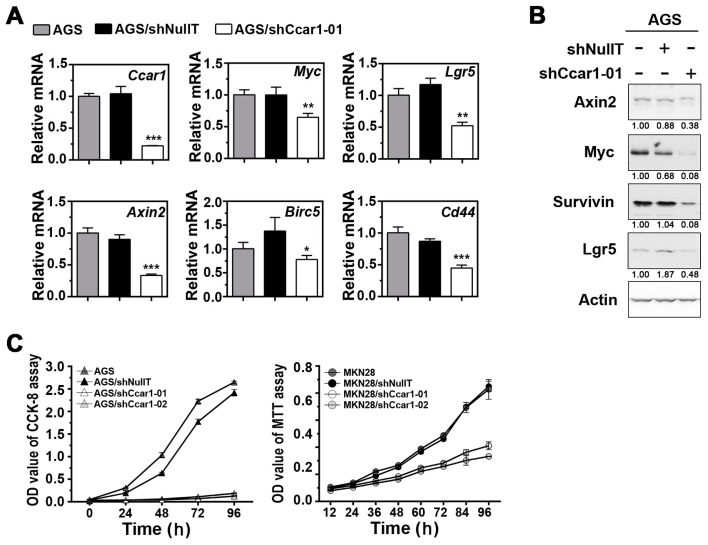
Cell cycle and apoptosis regulator 1 (CCAR1) knockdown affects cell growth. (**A**) The levels of the Wnt signaling downstream transcripts were quantified by real-time PCR using primers specific for the indicated genes. The results shown are normalized to the level of *Actb* mRNA; (**B**) Expression of Axin2, Myc, Survivin, and Lgr5 in AGS cells without infection, infected with control shRNA lentiviruses (shNullT), and infected with CCAR1-specific shRNA lentiviruses, were analyzed by western blot analysis. The density value of each band was normalized to Actin signal intensities and was expressed relative to the control (shown below each lane); (**C**) The growth curves of AGS/MKN28 cell variants with down-regulated CCAR1 were determined. The proliferation of AGS (**left**) and MKN28 (**right**) cell variants were monitored with MTT assay and their growth curves were plotted. Data are presented as the mean with error bars representing the S.D. (* *p* < 0.05; ** *p* < 0.01, *** *p* < 0.001).

Since studies have shown that the deregulation of many Wnt signaling components contributes to the development of gastric cancer, it is of interest to investigate whether CCAR1, a key coactivator of β-catenin, plays a role in the neoplastic transformation of gastric cancer. Lentiviral vectors expressing shRNA were used to reduce endogenous levels of CCAR1 in AGS and MKN28 cells. As shown in Figure 1A,B, the reduction of CCAR1 had a significant impact on the expression of several Wnt signaling target genes, such as *Axin2*, *Lgr5*, *CD44*, *Birc5*, and *c-myc*. This result confirms that CCAR1 does play a role in facilitating β-catenin in activating its downstream target genes. Interestingly, Lgr5 and CD44 are two known stem cell markers, suggesting that CCAR1 may also be important for the maintenance of gastric cancer stem cells. We further assessed the role of CCAR1 in the growth of gastric cancer cells. The growth curve of the lentivirus-infected cells demonstrated that, when CCAR1 was suppressed by either CCAR1-specific shRNA (shCCAR1-01 or shCCAR1-02) in AGS and MKN28, the growth of these two cell lines was significantly inhibited, when compared to either the untreated cells or the cells treated with shNullT (Figure 1C).

### 2.2. Suppression of CCAR1 Induces Apoptotic Cell Death in Gastric Cancer Cells

**Figure 2:**
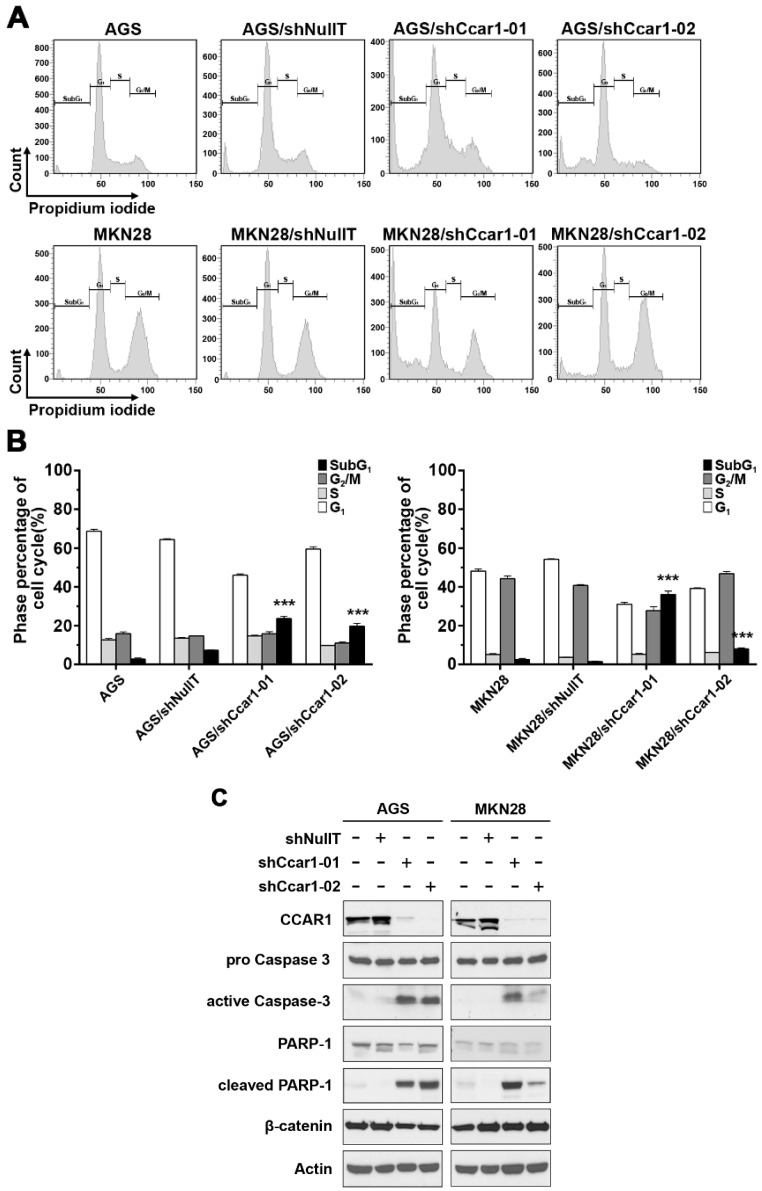
Suppression of CCAR1 induces apoptotic cell death in gastric cancer cells. (**A**) Cell cycle distribution of propidium iodide (PI)-labeled cells was analyzed by flow cytometric analyses. The peaks in the illustration correspond to the subG_1_, G_1_, S, and G_2_/M phases of the cell cycle; (**B**) Statistical analysis of cell cycle phase distribution. Data are presented as means ± SD of three independent tests. *** *p* < 0.001 versus control; (**C**) Expression of the apoptosis-related proteins, poly (ADP-ribose) polymerase 1 (PARP-1) and Caspase-3, and their cleaved patterns in gastric cancer cell lines (AGS and MKN28) without infection, infected with control shRNA lentiviruses (shNullT), and infected with two separate CCAR1-specific shRNA lentiviruses, were analyzed by western blot analysis. Actin was used as the loading control.

To further elucidate the mechanism of the suppressed cell growth caused by the knockdown of CCAR1, the lentivirus-infected cells were subjected to flow cytometry. When compared to the control group, more cells appeared in the sub-G_1_ phase when the cells’ endogenous CCAR1 were suppressed by shCCAR1-01 and shCCAR1-02: for AGS cells, the percentage of cells in the sub-G_1_ phase was up from 7.33% ± 0.21% (shNullT) to 23.63% ± 1.26% (shCCAR1-01) and 19.73% ± 1.40% (shCCAR1-02) when CCAR1 was suppressed; for MKN28 cells, the percentage of cells in the sub-G_1_ phase was up from 1.40% ± 0.17% (shNullT) to 36.03% ± 1.78% (shCCAR1-01) and 7.97% ± 0.59% (shCCAR1-02) when CCAR1 was suppressed (Figure 2A,B). This result indicates that CCAR1 is required for the survival of these cells. We further confirm this hypothesis by examining two apoptotic markers in the treated gastric cancer cells. As shown in Figure 2C, an increase of two apoptotic markers, cleaved PARP and active caspase 3, was observed in CCAR1-suppressed cells.

### 2.3. CCAR1 Mediates the Invasive Characters of Gastric Cancer Cells

**Figure 3:**
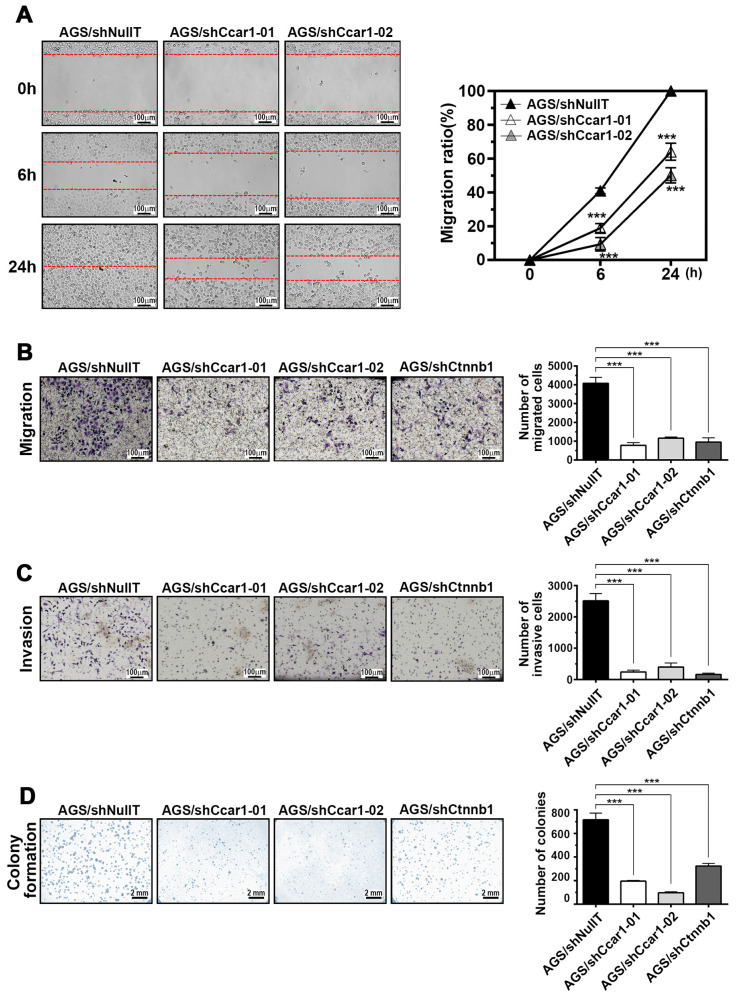
CCAR1 regulates cancer cell migration and invasion. The changes of cell colony formation, migration, and invasion ability affected by infected with CCAR1-specific shRNA lentiviruses were determined by the wound healing assay (**A**); transwell migration assay (without Matrigel) (**B**); invasion assay (with Matrigel) (**C**); and colony formation assay (**D**). Data are presented as the mean with error bars representing the S.D. (*** *p* < 0.001).

Besides examining the effects of reduced-CCAR1 expression on the growth of gastric cancer cells, we also investigated CCAR1’s functions on other characteristics of gastric cancer cells. In the wound-healing assay, suppression of CCAR1 expression apparently reduced the cell migration ability of AGS cells: while shNullT-infected cells nearly closed the wound at 24 h after scratching, shCCAR1-infected cells were unable to heal the wound (Figure 3A). Similarly, the Transwell migration assay showed that the number of migrating cells in the shCCAR1-infected group was significantly lower than that in the shNullT-infected group, indicating a decreased migratory ability following CCAR1 downregulation (Figure 3B). Next, we investigated the effect of CCAR1 on the invasion of AGS cells using the Transwell invasion assay. When compared with the shNullT-infected group, we found that CCAR1 knockdown significantly reduced the number of invading AGS cells (Figure 3C). These results indicate that CCAR1 promotes cell migration and invasion in gastric cancer cells. Also, as shown in Figure 3D, in the colony formation assay, much fewer colonies were observed in CCAR1-supressed cells than in control cells.

### 2.4. CCAR1 Is Required for the Expansion of the Gastric Cancer Xenograft in the Animal Model

**Figure 4:**
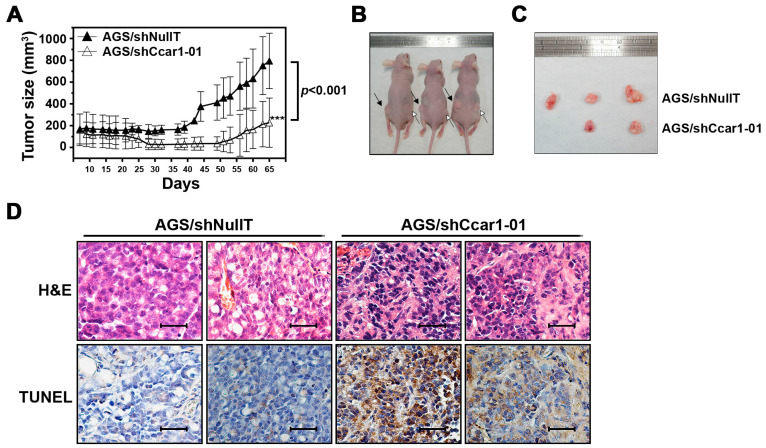
Effects of CCAR1 knockdown on xenograft gastric cancer formation. (**A**) Control or -shCCAR1-expressing AGS cells were injected subcutaneously into athymic nude mice to establish tumors. Tumor sizes were measured and depicted as tumor volume, and tumor growth curves were plotted accordingly. The symbol *** indicates *p* < 0.001; (**B**) Representative images showing subcutaneously injected xenograft tumors in nude mice at day 65 post subcutaneous injection. Black arrows indicate xenograft tumors derived from Control AGS cells, and white arrows indicate xenograft tumors derived from CCAR1 shRNA-expressing AGS cells; (**C**) Representative images showing respective xenograft tumors at day 65 post subcutaneous injection (*n* = 3 per group). Data are presented as the mean with error bars representing the S.D; (**D**) Representative images of H&E and terminal deoxynecleotidyl transferase-mediated dUTP nick-end-labeling (TUNEL) staining (brown) of xenografts. Scale bar: 40 µm.

To investigate whether the effect of suppressed CCAR1 on gastric cancer cells could be extended to in vivo conditions, we used a mouse xenograft model to examine the tumorigenic capabilities of cells in vivo. CCAR1-knockdown or control AGS cells (5 × 10^6^) were injected into nude mice. As shown in Figure 4A–C, tumors produced by the control cells displayed fast growth and reached a size of 800 mm^3^ in nine weeks, following the injection. On the other hand, tumors produced by CCAR1-suppressed cells grew much slower and reached a size of only 200 mm^3^. The apoptosis of the grafted tumors was further examined by the terminal deoxynecleotidyl transferase-mediated dUTP nick-end-labeling (TUNEL) assay. Figure 4D shows that the apoptosis was significantly higher in the tumors that were treated with shCCAR1, supporting the notion that reduced CCAR1 expression may induce apoptosis in gastric cancer cells.

### 2.5. The Expression of CCAR1 Is Significantly Stronger in Human Gastric Cancer Tissues Than Their Adjacent Normal Parts

**Figure 5:**
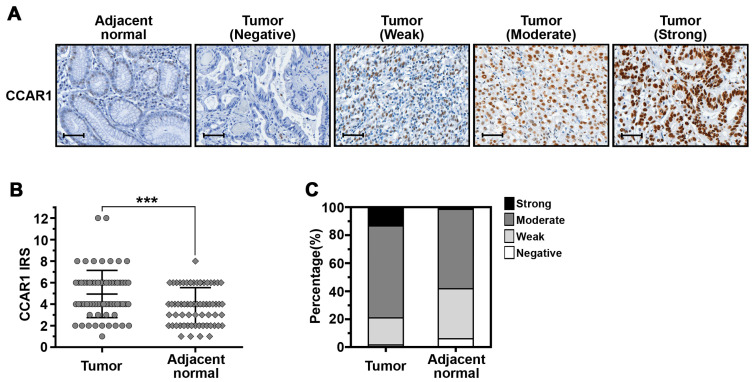
CCAR1 expression in human gastric cancer and matched adjacent normal tissues. (**A**) Representative images of immunohistochemical (IHC) staining for CCAR1 on the sections of gastric cancer tissues and their normal mucosa are shown. Scale bar: 40 μm (**B**) Scatter plot depicting CCAR1 levels as assessed by IRS (0–1 = negative staining; 2–3 = weak staining; 4–6 = moderate staining; 8–12 = strong staining) in tumors and adjacent normal tissues. Data are presented as the mean with error bars representing the S.D. The *p*-value was determined by an unpaired test. The symbol *** indicates *p* < 0.001; (**C**) Bar charts showing the percentage of cases that demonstrated staining with different levels of CCAR1 in tumors and adjacent normal tissues.

**Figure 6:**
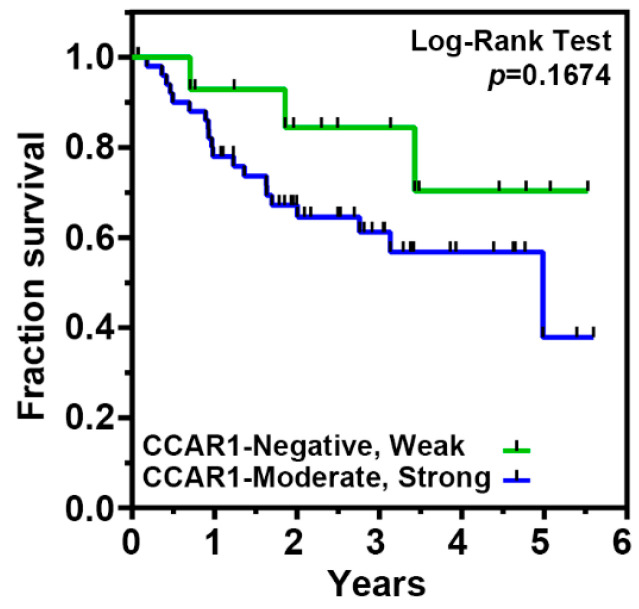
The relationship between CCAR1 expression and the outcome of patients with gastric cancer. Kaplan-Meier survival curves of patients with different CCAR1 expression.

Previous studies have shown that CCAR1 is a novel component of Wnt/β-catenin signaling, that plays an important role in the transcriptional regulation by β-catenin and which is involved in the neoplastic transformation of human colorectal cancer cells [19]. To investigate whether the expression of CCAR1 participates in the occurrence of gastric cancer, we analyzed the expression level of the CCAR1 protein by immunohistochemical (IHC) staining, on a tissue microarray (TMA) containing matched adjacent normal mucosa and gastric cancer tissue (*n* = 67) (Figure 5A). CCAR1 protein staining was quantified according to an adapted immunoreactive score (IRS) [20]. As shown in Figure 5B, the CCAR1 protein expression level was significantly higher in tumors when compared with paired normal tissues (*p* < 0.001). High-level protein expression (moderate and strong staining) of CCAR1 was observed in 79.1% (53/67) of tumors and 58.2% (39/67) of paired controls (Figure 5C). The Fisher’s exact test also demonstrated that significantly more primary tumor samples exhibited moderate/strong expression, when compared to their adjacent normal mucosa (*p* = 0.005) (Table 1). However, CCAR1 expression was not associated with age, gender, tumor stage, depth of invasion, lymph node metastasis, distant metastasis, and *H. pylori* infection (Table 2). Furthermore, the log-rank test was performed to examine the association between CCAR1 expression and overall patient survival, and the corresponding Kaplan-Meier plot is shown in Figure 6. Over the short 25.4-month median follow-up period, we observed that gastric cancer patients with moderate/strong CCAR1 expression had a poorer prognosis than those with negative/weak CCAR1 expression, but did not meet statistical significance.

## 3. Discussion

Cancer stem cells exist as a very small faction of a certain tumor and share many properties with somatic stem cells. Increasing evidence indicates that the presence of CSCs may determine the tumorigenesis, invasive growth, and metastasis of a tumor. Since the Wnt signaling pathway has been shown to maintain the self-renewal capacity of epithelial stem cells in the gastrointestinal tract, interrupting the Wnt signaling pathway may be effective in suppressing the expansion of gastric cancer stem cells. Here, we show that a component of the Wnt signaling pathway, CCAR1, plays a critical role in the tumorigenesis of gastric cancer. CCAR1 appears to be a biphasic regulator of cell growth and apoptosis. On the one hand, in breast cancer cells, CCAR1 is required for apoptosis induced by drugs such as CD437, Adriamycin, and Etoposide. On the other hand, CCAR1 is also involved in estrogen receptor signaling and promotes the growth of MCF-7 breast cancer cells in response to estradiol (E2) treatment. It appears that CCAR1 may exert different actions upon different cellular contents. Previously, Ou et al. have shown that CCAR1 interacts with β-catenin to activate its downstream target genes in HT29 colon carcinoma cells [19]. However, the reduced CCAR1 expression does not affect the growth of HT29 cells, but only affects the colony formation ability of HT29 cells. In this study, we knockdown the expression of CCAR1 in two gastric cancer cell lines using shRNA. Consequently, the expression of several β-catenin target genes decreases, including *Axin2*, *Lgr5*, *Cd44*, *Birc5*, and *c-myc*, indicating that CCAR1 functions in the same way in gastric cancer cells as it does in colon carcinoma cells. However, the growth of AGS and MKN28 cells was significantly retarded when the expression of CCAR1 was suppressed. From further examination, we found that the suppression of CCAR1 induced apoptosis in AGS and MKN28 cells, which could be verified by the presence of active caspase 3 and cleaved PARP1. Whether the apoptosis is induced by the reduced expression of *Birc5* (surviving), remains to be elucidated. Besides regulating cell growth, CCAR1 also functions as a mediator of oncogenic transformation in gastric cancer cells. Three different assays were separately performed in AGS and MKN28 cells to evaluate the importance of CCAR1 in regulating these cells’ invasive characters. The results of all the assays indicate that CCAR1 is essential for the migration and invasion of gastric cancer cells, and CCAR1 may be more important in the initiation and progression of gastric cancer than in that of colon cancer. Although β-catenin has been thought to mediate the epithelial-mesenchymal transition (EMT), the downstream target genes of CCAR1 and β-catenin responsible for this process are still under investigation. Interestingly, among the genes that are regulated by CCAR1, *Lgr5* and *CD44* are two stem cell surface markers, *Birc5* (surviving) mediates cell survival, and *c-myc* promotes cell proliferation. The altered expression of these target genes may reflect the physiological functions of CCAR1 in gastric cancer cells, which are to maintain stem cell characteristics and suppress apoptosis. Therefore, it is not surprising that the subcutaneous injection of CCAR1-suppressed AGS cells into nude mice resulted in smaller tumors than an injection with control AGS cells.

As mentioned above, the Wnt signaling pathway plays a crucial role in regulating proliferation, stem cell maintenance, and homeostasis in normal gastric mucosa. It is recognized that the dysregulation of the Wnt pathway plays a critical role in the development of human cancer. Indeed, different components of the Wnt pathway have been shown to be mutated or dysregulated in gastric cancer [21], including Wnt1 [22], SFRPs [23], Axin1 and Axin2 [24], APC [25,26], GSK3-β [27], and β-catenin [28]. For correcting the dysregulation of the Wnt pathway, it would be most effective to target the molecules located relatively downstream of the pathway. Thus, based on our study, we speculate that CCAR1 may serve as a good therapeutic target for treating gastric cancer patients.

## 4. Materials and Methods

### 4.1. Cell Lines, Reagents, and Patients

All the gastric cancer cell lines used in this work were cultured in Roswell Park Memorial Institute (RPMI) 1640 (Thermo Fisher Scientific, Waltham, MA, USA), supplemented with 10% fetal bovine serum, 100 units/mL penicillin, and 100 mg/mL streptomycin (Thermo Fisher Scientific), in a 5% CO_2_ atmosphere at 37 °C. Formalin-fixed and paraffin-embedded tissue samples were obtained from 67 patients with a mean age of 68.1 ± 10.0 years who had received a curative gastrectomy between 2010 and 2014 at Chang Gung Memorial Hospital, Chiayi, Taiwan. The acquisition and use of clinical specimens in this study were approved by the Institutional Review Board of Chang Gung Medical Foundation (IRB approval number: 102-1996B, approval date: 16 July 2013).

### 4.2. Tissue Microarray

Matched pairs of paraffin-embedded gastric cancer samples and adjacent gastric tissues were used for the construction of tissue microarray (TMA). Briefly, Hematoxylin and Eosin-stained sections were made from each selected donor block to define representative tissue regions. Tissue cylinders (1.5 mm in diameter) were then punched from the region of the donor block and were transferred to an 18 mm × 30 mm paraffin block to produce the TMA block, with the use of an automatic tissue microarray instrument (AutoTiss 1000, Ever BioTechnology, Taipei, Taiwan). The resulting TMA block was cut into 5-µm sections that were placed on 3-amminopropylltriethoxysillane-coated glass slides using the sliding microtome (Leica SM 2000 R, Leica Biosystems, Wetzlar, Germany). Sections from the TMA blocks were then used for immunohistochemical analysis.

### 4.3. Immunohistochemical Staining

For detection of the Wnt signaling components in cancer tissues, the paraffin-embedded gastric cancer tissues (5-μm-thick sections) were de-waxed with xylene and rehydrated in graded alcohol. The deparaffinized sections were then heated in 10 mM citrate buffer (pH 6.0) for 10 min for antigen-retrieval. Following treatment with 3% H_2_O_2_ at room temperature for 30 min, slides were then washed, blocked with 100% normal goat serum in PBST containing 0.05% Tween-20, and incubated with each primary antibody at 4 °C overnight. Immunohistochemical staining was performed using the SuperPicture™ 3rd Gen IHC Detection Kit (Thermo Fisher Scientific). Primary antibodies were used following the manufacturers’ instructions and were tested for the best dilution. The immunostained sections were then counterstained with hematoxylin, dehydrated, and mounted. The percentages of cells with positive staining were used as immunohistochemical scores. Scores for a percentage of CCAR1-positive cells and scores for expression intensities were multiplied to calculate an immunoreactive score (IRS). The apoptosis of xenograft tissues was examined by a TUNEL assay, with the use of an In Situ Cell Death Detection kit (Roche, Basesl, Switzerland).

### 4.4. Lentiviral Production and Infection

The plasmids used for producing the shCcar1 lentivirus (pLKO.1-shCcar1-01: TRCN0000056003 and pLKO.1-shCcar1-02: TRCN0000376588) and the lentivirus carrying the scrambled shRNA (pLKO.1-shNullT) were obtained from the National RNAi Core Facility located at the Institute of Molecular Biology/Genomic Research Center, Academia Sinica in Taiwan. Plasmid DNA was transfected into HEK293T cells for the production of lentiviral particles, according to established protocols. For lentiviral transduction, target cells were seeded in appropriate vessels. During the following day, media was replenished and supplemented with 8 μg/mL polybrene (Sigma, St. Louis, MO, USA) and lentivirus-containing medium. After 24–48 h, cells were incubated with new media. In all experiments, scramble shRNA (shNullT) was used as a negative control.

### 4.5. Colony Formation Assay

Cells infected with shRNAs after 48 h were seeded in 6-well plates at a density of 2000 cells/well and incubated for four days. The colonies were fixed with methanol at room temperature for 10 min, stained with 0.4% crystal violet for 10 min, and finally, positive colony formation (more than 50 cells/colony) was counted, and the colony formation rate was calculated.

### 4.6. Wound Healing and Transwell Migration Assay

Cell migration was analyzed using a wound-healing and transwell-migration assay. For the wound-healing assay, infected cells were cultured in six-well plates. When the cells were up to 90% confluence, three scratch wounds across each well were made using a P-200 pipette tip. Plates were washed twice with PBS to remove detached cells and incubated with the complete growth medium without FBS, and the wound-closing procedure was observed for 48 h. Photographs were taken at 0, 6, 24, and 48 h, respectively. The result was expressed as a migration rate: the area covered by the migrating cells (time)/the wound area (0 h). For the transwell-migration assay, cells were serum-starved for 6 h and suspended in serum-free RPMI 1640. The cell suspension (5 × 10^4^ cells) was added to the upper chamber of Millicell^®^ tissue culture plate well inserts (Merk Millipore, Billerica, MA, USA) without Matrigel, and was incubated for 24 h with medium containing 10% FBS in the bottom of the chamber. Residual cells on the upper side of chambers were removed by scraping with cotton swabs, and the cells that attached to the lower side of the membrane were fixed with methanol for 10 min and stained with 0.4% crystal violet/50% methanol for 10 min. Cell numbers were counted in four random fields (×100) per filter and photographs were taken by microscopy.

### 4.7. Invasion Assay

For the invasion assay, cells were serum-starved for 6 h and suspended in serum-free RPMI 1640. The cell suspension (5 × 10^4^ cells) was added to the upper chamber of BD BioCoat Matrigel Invasion Chambers (Becton Dickinson, Franklin Lakes, NJ, USA) and was incubated for 24 h with medium containing 10% FBS in the bottom of the chamber. Residual cells on the upper side of the chambers were removed by scraping with cotton swabs, and the cells that attached to the lower side of the membrane were fixed with methanol for 10 min and stained with 0.4% crystal violet/50% methanol for 10 min. Cell numbers were counted in three random 100× fields per filter and photographs were taken by microscopy.

### 4.8. RNA Isolation and RT-Quantitative PCR

The total RNA was extracted with a TRIzol reagent, according to the manufacturer’s instructions (Thermo Fisher Scientific), and treated with DNaseI (Epicentre, Madison, WI, USA) to remove residual genomic DNA. The cDNA synthesis was performed using MMLV high-performance reverse transcriptase (Epicentre) at 37 °C for 80 min, in a final volume of 20 μL, according to the manufacturer’s instructions. Quantitative PCR was performed using SYBR^®^ Select Master Mix in the CFX96 Touch™ Real-Time PCR Detection System (BIO-RAD Laboratories, Hercules, CA, USA). The sequences of the primers were as follows:

*Ccar1* forward 5′-CTGATGGCTAGCCCTAGTATGGA-3′,
*Ccar1* reverse 5′-TGCCTTTCATGCCCACTAAAA-3′,
*Axin2* forward 5′-GGAATCATTCGGCCACTGTT-3′,
*Axin2* reverse 5′-TGGACACCTGCCAGTTTCTTT-3′,
*Cd44* forward 5′-ACCTTTCCCCCACCAGCTA-3′,
*Cd44* reverse 5′-GCTATGGAAGGGCAAAATGG-3′,
*Lgr5* forward 5′-CACTTGCTTTCCAAATGGGTTT-3′,
*Lgr5* reverse 5′-ATCACAGCCTCTACCTAGCAATGTAG-3,
*Myc* forward 5′-GGCCCCCAAGGTAGTTATCC-3′,
*Myc* reverse 5′-GTTTCCGCAACAAGTCCTCTTC-3′,
*Birc5* forward 5′-GACCCACTTATTTCTGCCACATC-3′,
*Birc5* reverse 5′-GAGTACAGAGGCTGGAGTGCATT-3′,
*Actb* forward 5′-TGGCATTGCCGACAGGAT-3′,
*Actb* reverse 5′-CGCTCAGGAGGAGCAATGAT-3′.

The expression levels of the genes analyzed were normalized to the housekeeping gene *Actb*.

### 4.9. Tumorigenicity in Nude Mice

Eight-week-old male athymic nude mice were purchased from the National Laboratory Animal Center (Tainan, Taiwan). AGS cells (5 × 10^6^/mouse) were mixed with an equal volume of Matrigel (Becton Dickinson), suspended in 100 µL of PBS, and injected subcutaneously into the posterior leg of athymic nude mice. The mice were randomly divided into two groups: scramble shRNA (shNullT) and shRNA-Ccar1, with three mice in each group. Tumor length (L) and width (W) were measured every 2–3 days, and tumor volume was calculated using the equation: volume = 1/2 × length × width^2^. The mice were sacrificed about 65 days after inoculation. Tumors were dissected and harvested for pathological analysis. All of the animal experiments were performed with the approval of the Institutional Animal Care and Use Committee at our Laboratory Animal Center, Department of Medical Research, Chang Gung Memorial Hospital at Chiayi (IACUC no: 2013121709, approval date: 29 January 2014).

### 4.10. Cell Proliferation Assay

Cell viability was evaluated by a 3-[4,5-dimethylthiazol-2-yl]-2,5-diphenyl tetrazolium bromide (MTT) (Sigma) assay. The cells of test groups were plated at a density of 2 × 10^3^ cells/well in 96-well culture plates and were analyzed at different time points. Then, MTT (50 µL, 5 mg/mL) was added to each well and incubated at 37 °C for 3 h. The reaction was terminated by replacing the MTT-containing medium with 500 μL of dimethyl sulfoxide (Sigma), and the formazan salts were dissolved by gentle shaking for approximately 5 min at room temperature. The optical density (OD) of each well was measured at 545 nm using a microplate reader. Each assay was completed in triplicate wells, and each experiment was repeated three times.

### 4.11. Statistical Analysis

Statistical analyses were performed using SPSS 12.0 software (SPSS Inc., Chicago, IL, USA). Results were shown as the mean ± standard deviation (SD). The associations between the expression status and pathological parameters were analyzed using the Fisher’s exact tests. Survival curves were fitted with the Kaplan-Meier method and the differences in survival were assessed by the log-rank test. Differences between experimental groups were assessed by the Student’s *t*-test or one-way ANOVA. A two-tailed value of *p* < 0.05 was deemed as statistically significant.

## Figures and Tables

**Table 1:** The Fisher’s exact test of the IHC staining of 67 cases of the paired gastric cancer specimens on TMA chips.

Samples	CCAR1 Expression				*p*-Value
	Negative (%)	Weak (%)	Moderate (%)	Strong (%)	
Adjacent normal	4 (6.0%)	24 (35.8%)	38 (56.7%)	1 (1.5%)	0.005
Tumor	1 (1.5%)	13 (19.4%)	44 (65.7%)	9 (13.4%)	

**Table 2:** Association between clinicopathological features and CCAR1 expression.

Features	CCAR1 Expression of Tumor Part		*p*-Value
	Negative, Weak (%)	Moderate, Strong (%)	
Gender			
Male	11 (23.4%)	36 (76.6%)	0.528
Female	3 (15.0%)	17 (85.0%)	
Age			
<65 years	5 (23.8%)	16 (76.2%)	0.692
≥65 years	9 (19.6%)	37 (80.4%)	
Pathological Stage			
I + II	7 (24.1%)	22 (75.9%)	0.569
III + IV	7 (18.4%)	31 (81.6%)	
Depth of invasion (pT)			
T1 + T2	5 (23.8%)	16 (76.2%)	0.751
T3 + T4	9 (19.6%)	37 (80.4%)	
Lymph nodes metastasis			
Negative	6 (26.1%)	17 (73.9%)	0.450
Positive	8 (18.2%)	36 (81.8%)	
Distant metastasis			
Negative	11 (19.3%)	46 (80.7%)	0.425
Positive	3 (30.0%)	7 (70.0%)	
*H. pylori*			
Negative	3 (13.0%)	20 (87.0%)	0.349
Positive	11 (25.0%)	33 (75.0%)	
