# Correction: Chang et al. Inhibition of CCAR1, a Coactivator of β-Catenin, Suppresses the Proliferation and Migration of Gastric Cancer Cells. *Int. J. Mol. Sci.* 2017, *18*, 460

**DOI:** 10.3390/ijms27114737

**Published:** 2026-05-25

**Authors:** Te-Sheng Chang, Kuo-Liang Wei, Chung-Kuang Lu, Yi-Hsing Chen, Ying-Tung Cheng, Shui-Yi Tung, Cheng-Shyong Wu, Ming-Ko Chiang

**Affiliations:** 1 Department of Gastroenterology and Hepatology, Chang Gung Memorial Hospital, Chiayi 61303, Taiwan; cgmh3621@adm.cgmh.org.tw (T.-S.C.); wkliang@adm.cgmh.org.tw (K.-L.W.); cmusickimo@yahoo.com.tw (C.-K.L.); a12509@cgmh.org.tw (Y.-H.C.); ma1898@adm.cgmh.org.tw (S.-Y.T.); gi0005@adm.cgmh.org.tw (C.-S.W.); 2 College of Medicine, Chang Gung University, Taoyuan 33302, Taiwan; 3 Department of Life Science, National Chung Cheng University, Chiayi 62102, Taiwan; s26136282@livemail.tw

In the original publication [1], an error occurred during the preparation of Figure 3. In panel C, the image for AGS/shCcar1-02 was inadvertently duplicated in the position intended for AGS/shCcar1-01. The authors have provided a corrected version of Figure 3. The authors state that the scientific conclusions are unaffected. This correction was approved by the Academic Editor. The original publication has also been updated.

## Figures and Tables

**Figure 3:**
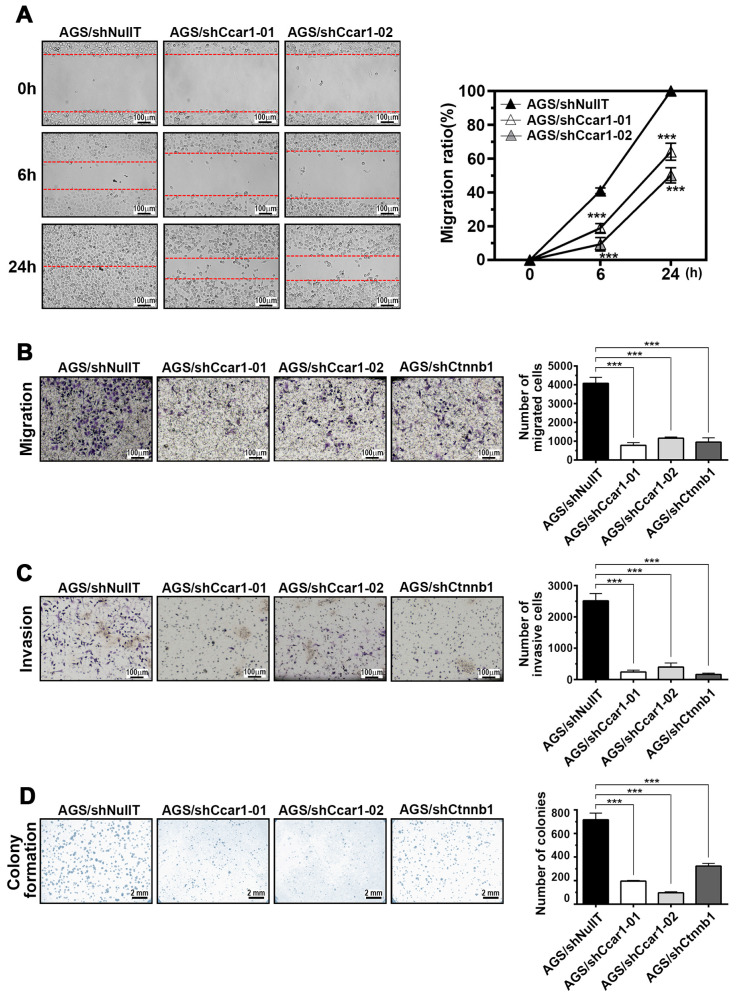
CCAR1 regulates cancer cell migration and invasion. The changes of cell colony formation, migration, and invasion ability affected by infected with CCAR1-specific shRNA lentiviruses were determined by the wound healing assay (**A**); transwell migration assay (without Matrigel) (**B**); invasion assay (with Matrigel) (**C**); and colony formation assay (**D**). Data are presented as the mean with error bars representing the S.D. (*** *p* < 0.001).
